# An intranasal stringent response vaccine targeting dendritic cells as a novel adjunctive therapy against tuberculosis

**DOI:** 10.3389/fimmu.2022.972266

**Published:** 2022-09-16

**Authors:** Styliani Karanika, James T. Gordy, Pranita Neupane, Theodoros Karantanos, Jennie Ruelas Castillo, Darla Quijada, Kaitlyn Comstock, Avinaash K. Sandhu, Aakanksha R. Kapoor, Yinan Hui, Samuel K. Ayeh, Rokeya Tasneen, Stefanie Krug, Carina Danchik, Tianyin Wang, Courtney Schill, Richard B. Markham, Petros C. Karakousis

**Affiliations:** ^1^ Division of Infectious Diseases, Department of Medicine, The Johns Hopkins Hospital, Baltimore, MD, United States; ^2^ Center for Tuberculosis Research, Department of Medicine, Johns Hopkins University School of Medicine, Baltimore, MD, United States; ^3^ W. Harry Feinstone Department of Molecular Microbiology and Immunology, Johns Hopkins Bloomberg School of Public Health, Baltimore, MD, United States; ^4^ Division of Hematological Malignancies, Department of Oncology, Sidney Kimmel Comprehensive Cancer Center, Johns Hopkins University Hospital, Baltimore, MD, United States

**Keywords:** *Mycobacterium tuberculosis*, tuberculosis DNA vaccines, persistence, stringent response, immunotherapy, intranasal route, T cells

## Abstract

Lengthy tuberculosis (TB) treatment is required to overcome the ability of a subpopulation of persistent *Mycobacterium tuberculosis* (*Mtb*) to remain in a non-replicating, antibiotic-tolerant state characterized by metabolic remodeling, including induction of the Rel_Mtb_-mediated stringent response. We developed a novel therapeutic DNA vaccine containing a fusion of the *rel_Mtb_* gene with the gene encoding the immature dendritic cell-targeting chemokine, MIP-3α/CCL20. To augment mucosal immune responses, intranasal delivery was also evaluated. We found that intramuscular delivery of the *MIP-3α*/*rel_Mtb_* (fusion) vaccine or intranasal delivery of the *rel_Mtb_* (non-fusion) vaccine potentiate isoniazid activity more than intramuscular delivery of the DNA vaccine expressing *rel_Mtb_* alone in a chronic TB mouse model (absolute reduction of *Mtb* burden: 0.63 log_10_ and 0.5 log_10_ colony-forming units, respectively; P=0.0002 and P=0.0052), inducing pronounced *Mtb*-protective immune signatures. The combined approach involving intranasal delivery of the DNA *MIP-3α*/*rel_Mtb_* fusion vaccine demonstrated the greatest mycobactericidal activity together with isoniazid when compared to each approach alone (absolute reduction of *Mtb* burden: 1.13 log_10_, when compared to the intramuscular vaccine targeting *rel_Mtb_* alone; P<0.0001), as well as robust systemic and local Th1 and Th17 responses. This DNA vaccination strategy may be a promising adjunctive approach combined with standard therapy to shorten curative TB treatment, and also serves as proof of concept for treating other chronic bacterial infections.

## Introduction

Tuberculosis (TB) is a major cause of morbidity, and the second leading infectious killer after COVID-19 worldwide (1). The current six-month regimen, consisting of isoniazid (INH), rifampin, pyrazinamide and ethambutol, has high efficacy against drug-sensitive TB, but its length and complexity contributes to treatment interruptions that jeopardize cure and promote drug resistance (2, 3). Although novel, treatment-shortening antibiotic regimens have shown promising results in international clinical trials (4, 5), the resources required for direct observation of daily treatment and the associated costs may still pose barriers to their implementation in TB-endemic countries. Recent work has focused on adjunctive, host-directed strategies to simplify and shorten the course of TB therapy (6).

The need for prolonged TB treatment is believed to reflect the unique ability of a subpopulation of *Mycobacterium tuberculosis* (*Mtb*) bacilli within the infected host to remain in a nonreplicating, persistent state (7) characterized by tolerance to first-line anti-TB drugs, like INH, which more effectively targets actively dividing bacilli (8–11). One of the key bacterial pathways implicated in antibiotic tolerance is the stringent response, which is regulated by the (p)ppGpp synthase/hydrolase, Rv2583c (Rel_Mtb_) (12, 13). Rel_Mtb_ deficiency results in defective *Mtb* survival under nutrient starvation (14) and in mouse lungs (15) and mouse hypoxic granulomas (16), reduced virulence in guinea pigs (17) and C3HeB/FeJ mice (18), and increased *Mtb* susceptibility to INH in mouse lungs (18), rendering Rel*_Mtb_* an attractive target for novel antitubercular therapies, including for drug-resistant TB (13).

We previously showed that intramuscular (IM) delivery of a DNA vaccine expressing *rel_Mtb_* enhanced the mycobactericidal activity of INH in a murine TB model (3, 19). In independent studies, our group has shown the enhanced efficacy of vaccines when the antigen of interest is fused to the gene encoding the chemokine Macrophage Inflammatory Protein-3 alpha/C-C Motif Chemokine Ligand 20 (MIP-3α/CCL20) (20–22). This chemokine targets the antigen of interest to immature dendritic cells (DCs) and, compared to vaccines without the MIP-3α component, has shown enhanced immune responses in both melanoma and malaria model systems (20–22). These earlier studies have also demonstrated that no host immune response is elicited to the autologous chemokine component of the vaccine (20–22). Since T-cell immunity is required to control *Mtb* infection (6), we hypothesized that fusion of *rel_Mtb_* to the chemokine gene MIP-3α (yielding *MIP-3α/rel_Mtb_* or “fusion vaccine”) would enhance the immunogenicity of the *rel_Mtb_* vaccine and further potentiate the mycobactericidal activity of INH *in vivo*.

Protection against pulmonary TB is associated with the ability of anti-*Mtb* T cells to exit the pulmonary vasculature and enter into the lung parenchyma and airways (23). Intranasal (IN) vaccination has been shown to promote recruitment of antigen-experienced T cells to these restricted lung compartments in contrast to parenteral immunization (23). Thus, we also hypothesized that IN administration of the vaccine expressing *rel_Mtb_* alone or the fusion vaccine would further augment T-cell responses within the lung, the primary site of *Mtb* infection.

Here, we present our bacteriological and immunological findings of a DNA vaccine expressing *rel_Mtb_* alone or the fusion construct, administered by the IM or IN route, in a murine model of chronic TB. Our results indicate that IM delivery of the *MIP-3α/rel_Mtb_* fusion vaccine or IN delivery of the *rel_Mtb_* vaccine yielded statistically equivalent improvement of mycobacterial outcomes compared to IM delivery of the *rel_Mtb_* vaccine. IN delivery of the *MIP-3α/rel_Mtb_* fusion vaccine (“optimized vaccination strategy”) yielded the highest additive therapeutic effect compared to each single novel approach alone.

## Methods

### Bacteria and growth conditions

Wild-type *Mtb* H37Rv was grown in Middlebrook 7H9 broth (Difco, Sparks, MD) supplemented with 10% oleic acid-albumindextrose- catalase (OADC, Difco), 0.2% glycerol, and 0.05% Tween-80 at 37°C in a roller bottle (3).

### Antigen preparation

The previously generated *rel_Mtb_* expression plasmid, pET15b[rel_Mtb_] (3, 19), was used for expression and purification of recombinant Rel_Mtb_ protein. *Escherichia coli* BL21 (DE3)

RP competent cells (Stratagene) were transformed with pET15b[rel_Mtb_]. Transformed bacteria were selected with ampicillin (100 mg/ml), and cloning was confirmed by DNA sequencing. Protein expression was performed using standard protocols and purification was performed using Ni-NTA Agarose (Qiagen). Recombinant Rel*_Mtb_* protein (87 kDa) was purified from the cell lysate using a Ni-NTA resin column. The purity was confirmed by SDS-PAGE gel and immunoblot analyses. The protein concentration was determined using a BCA protein assay with BSA as the standard (Thermo Fisher). Recombinant Rel_Mtb_ has been shown previously to retain (p)ppGpp synthesis and hydrolysis activities and can serve as an antigen to measure Rel_Mtb_-specific T-cell responses *ex vivo* (3, 19).

### DNA vaccines

**Figure 1:**
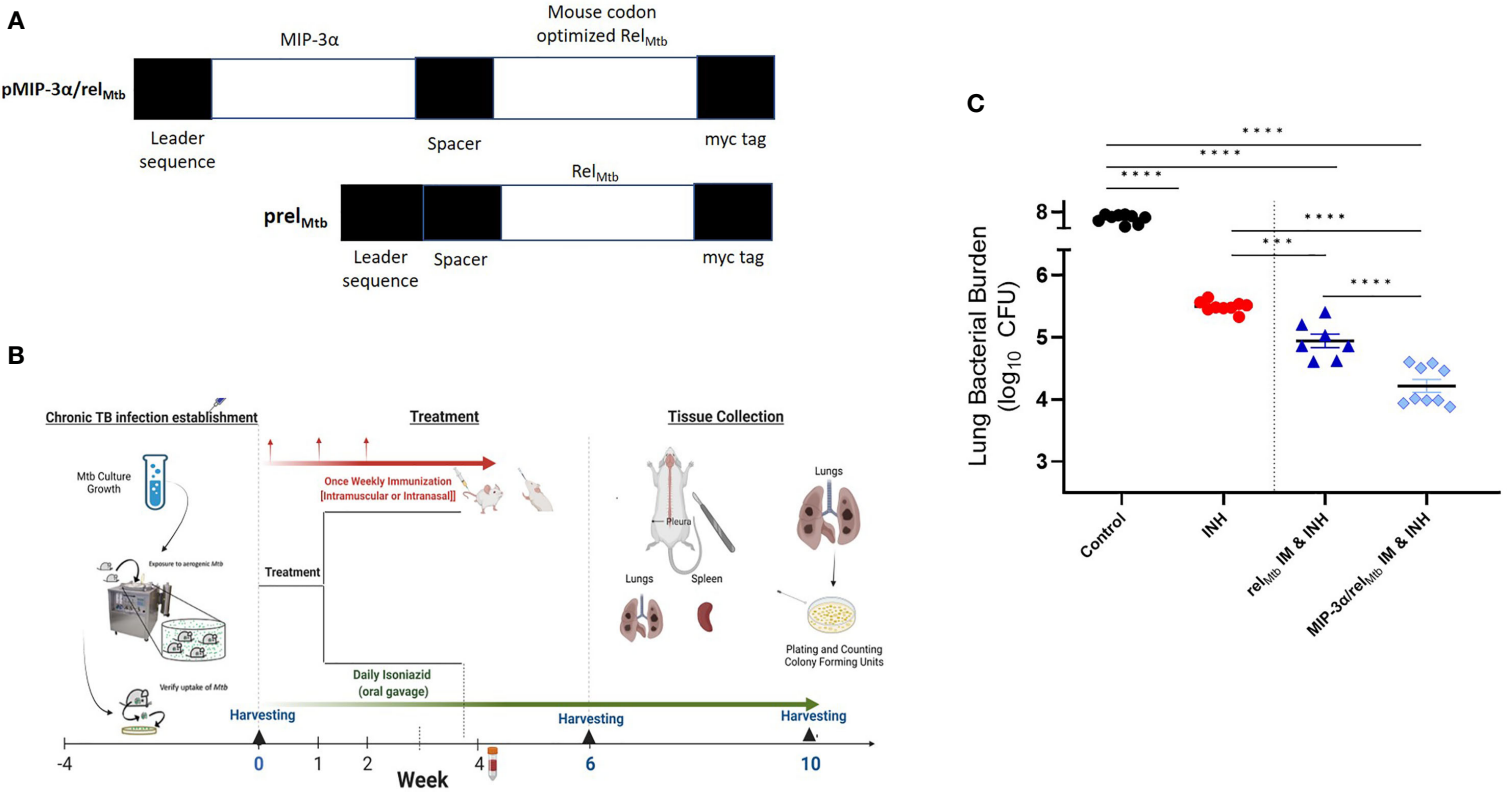
IM delivery of the *MIP-3α/rel_Mtb_* fusion vaccine increases the mycobactericidal activity of INH in a murine model of chronic TB. **(A)** Vaccination constructs; **(B)** Timeline of the *Mtb* challenge study; **(C)** Scatterplot of lung mycobacterial burden at 10 weeks after the primary vaccination per vaccination group. *Mtb*, *Mycobacterium tuberculosis*; IM, Intramuscular; CFU, colony-forming units; INH, Isoniazid. ***P < 0.001, ****P < 0.0001.

The plasmid pSectag2B encoding the full-length *rel_Mtb_* gene was used as the *rel_Mtb_* DNA vaccine (19). The *rel_Mtb_* gene was codon-optimized (Genscript) and fused to the mouse *MIP-3α* gene. The fusion product was cloned into pSectag2B, serving as the *MIP-3α/rel_Mtb_*, or “fusion” vaccine (**Figure 1A**, detailed sequence in **Supplementary Appendix**). Proper insertion was confirmed by sequencing and the expression of target genes was confirmed by transfection of 293T cells in lysates and supernatants. Vaccination plasmids were selected by ampicillin (100 μg/ml) and extracted from *E. coli* DH5-α (Invitrogen™ ThermoFisher Scientific, Waltham, MA) using Qiagen^®^ (Germantown, MD) EndoFree^®^ Plasmid Kits and were diluted with endotoxin-free 1xPBS.

### *Mtb* challenge study in mice

Seven to ten male and female C57BL/6 mice (8-10-week-old, The Jackson Laboratory) were aerosol-infected with ~100 bacilli of wild-type *Mtb* H37Rv using a Glas-Col Inhalation Exposure System (Terre Haute, IN). After 28 days of infection, the mice received INH in a concentration of 10 mg/kg dissolved in total volume of 100 μl of distilled water per mouse. INH was administered by esophageal gavage once daily (5 days/week) and mice were randomized to receive the *rel_Mtb_* vaccine or the fusion vaccine by the IM or IN route. The mice were vaccinated three times at one-week intervals. IM or IN delivery of each plasmid followed adequate anesthesia of mice by vaporized isoflurane. For IM vaccinations, each plasmid was injected bilaterally into the quadriceps femoris muscle of the mice (50 μL in each quadriceps), followed by local electroporation using an ECM830 square wave electroporation (EP) system (BTX Harvard Apparatus Company, Holliston, MA, USA), since EP can increase the antigen uptake up to 1000 times (24). Each of the two-needle array electrodes delivered 15 pulses of 72V (a 20-ms pulse duration at 200-ms intervals) (19). For IN vaccinations, each plasmid was administered into both nostrils (50 μL in each nostril) and mice were monitored in the upright position until complete recovery and vaccine absorption were assured. To compensate for the anticipated reduced plasmid uptake without EP, which cannot be used with the IN vaccination route, we increased the dose of the vaccine 10-fold. The mice were sacrificed 6 weeks and 10 weeks after treatment initiation. The spleens and left lungs were harvested and processed into single-cell suspensions. The cells were then filtered through a 70-mm nylon filter mesh to remove undigested tissue fragments and washed with complete RPMI medium.The right lungs were homogenized using glass homogenizers. Serial tenfold dilutions of lung homogenates in PBS were plated on 7H11 selective agar (BD) at the indicated time points. Plates were incubated at 37°C and colony-forming units (CFU) were counted 4 weeks later by at least 2 investigators (3, 19). All procedures were performed according to protocols approved by the Johns Hopkins University Institutional Animal Care and Use Committee.

### Immunogenicity studies in mice

Three to five male and female C57BL/6 mice (8-10-week-old, Charles River Laboratory) were randomized to receive the *rel_Mtb_* or the fusion DNA vaccine by the IM or IN route. The mice were sacrificed 6 weeks after the primary vaccination. Spleens, draining lymph nodes (LNs), lungs and peripheral blood mononuclear cells (PBMCs) were collected and processed into single-cell suspensions individually.

### Intracellular cytokine staining, flow cytometry analysis and fluorospot

Single-cell suspensions from spleens, draining LNs, lungs and PBMCs were prepared. Each tissue was stimulated individually with purified recombinant Rel_Mtb_ protein at 37°C (3, 19) for various time intervals, from 12 hrs (IFN-γ, IL-17α, IL-2) to 24 hrs (TNF-α), depending on the cytokine of interest. For Intracellular Cytokine Staining (ICS), GolgiPlug cocktail (BD Pharmingen, San Diego, CA) was added for an additional 4 hours after stimulation (total, 16 and 28 hours, respectively) and cells were collected using FACS buffer (PBS + 0.5% Bovine serum albumin (Sigma-Aldrich, St. Louis, MO), stained with Zombie NIR™ Fixable Viability Kit (Biolegend Cat. No.: 423105) for 30 min, and washed with PBS buffer. Surface proteins were stained for 20 min, the cells were fixed and permeabilized with buffers from Biolegend intracellular fixation/permeabilization set following manufacturer protocols (Cat. No. 421002), intracellular proteins were stained for 20 min, and samples were washed and resuspended with FACS buffer. The following anti-mouse mAbs were used for ICS: PercPCy5.5 conjugated anti-CD3 (Biolegend Cat. No 100217), FITC-conjugated anti-CD4 (Biolegend Cat. No 100405), Alexa700 conjugated anti-CD8 (Biolegend Cat. No. 155022), PECy7 conjugated anti-TNF-α, (Biolegend Cat. No. 506323), APC conjugated anti-IFN-γ, (Biolegend Cat. No. 505809), BV421 conjugated anti-IL-2, (Biolegend Cat. No 503825), PE conjugated anti-IL-17α (Biolegend Cat. No 506903). The Attune™ NxT (Thermo Fisher Scientific, Waltham, MA), and a BD™ LSRII flow cytometer was used. Flow data were analyzed by FlowJo Software (FlowJo 10.8.1, LLC Ashland, OR). Flow analysis included alive, gated, total T lymphocytes, including CD4+ and CD8+ T-cell subpopulations. For simplicity, the CD8+ subpopulation analysis is not reported if no substantial population to allow comparisons was detected (e.g., IL-17α). For FluoroSpot assays (**Supplementary Data**), kits with pre-coated plates for enumeration of cells secreting IFN-γ and IL-17A were purchased from Mabtech (Cat. No. FSP-414443-2). Spots were enumerated on an AID iSpot EliSpot/FluoroSpot Reader.

### Statistics

Pairwise comparisons of group mean values for log_10_ CFU (microbiology data) and flow cytometry data were made using one-way analysis of variance followed by Tukey’s multiple comparisons test. Prism 9.3 (GraphPad Software, Inc. San Diego, CA) was utilized for statistical analyses and figure generation. To illustrate the aggregate cytokine data per group (**Figure 4**), fraction of total analysis was used and is displayed in stacked bars. In **Figure 6**, cytokine data were normalized (the total sum of all the experimental groups= 100%, total absolute number of alive cells=30,000). All error bars represent the estimation of the standard error of the mean, and all midlines represent the group mean. A significance level of α ≤ 0.05 was set for all experiments.

## Results

### Intramuscular administration of *MIP-3α/rel_Mtb_* fusion vaccine increases the mycobactericidal activity of INH and elicits robust systemic Th1 responses in a murine model of chronic TB

**Figure 2:**
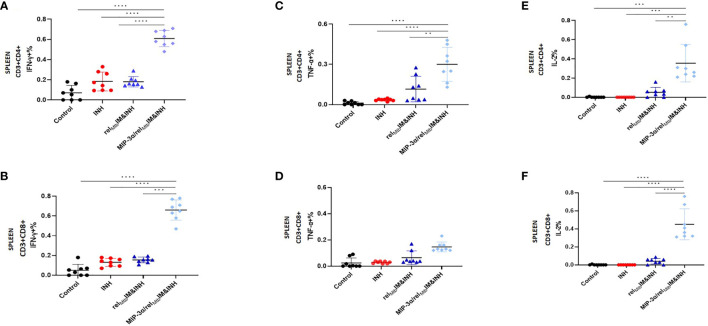
T-cell responses in murine spleens 6 weeks after *Mtb* challenge: IM vaccination with *MIP-3α*/*rel_Mtb_* elicits higher systemic Th1 response compared to IM vaccination with *rel_Mtb._* Rel_Mtb_-specific IFN-γproducing CD4+ T cells **(A)** and CD8+ T cells **(B)**; Rel_Mtb_-specific, TNF-α-producing CD4+ T cells **(C)** and CD8+ T cells **(D)**; Rel_Mtb_-specific IL-2-producing CD4+ T cells **(E)** and CD8+ T cells, **(F)** Flow cytometry-intracellular staining. IM, Intramuscular; IN, Intranasal. Y-axis scales are different among cytokines and between tissues in order to better demonstrate differences between groups where cytokine expression levels were lower. **P < 0.01, ***P < 0.001, ****P < 0.0001.

Four weeks after *Mtb* aerosol infection, C57BL/6 mice were treated daily with human-equivalent doses of oral INH for 10 weeks (**Figure 1B**). The *MIP-3α/rel_Mtb_* fusion vaccine [**Figure 1A**, detailed sequences are available in the **Supplementary Appendix**] was administered *via* the IM route weekly for 3 weeks. The original vaccination strategy (IM delivery of the *rel_Mtb_* vaccine), which previously demonstrated therapeutic adjunctive activity together with INH (3, 19), served as our baseline comparator in this study (“comparator” vaccine). One negative control group received no treatment, while another group received INH only. All vaccinated groups received INH in addition to the tested vaccines. Since DNA vaccination alone did not exhibit significant mycobactericidal activity in prior work (19), this group was not included in the present study. At 10 weeks after primary vaccination *via* the IM route, greater reduction in the lung mycobacterial burden was observed in the group receiving the fusion vaccine along with INH compared to that receiving the comparator vaccine with INH [absolute reduction of mycobacterial burden: 0.63 log_10_ CFU (P=0.0001), **Figure 1C**]. Relative to the *rel_Mtb_* vaccine, the *MIP-3α/rel_Mtb_* fusion vaccine elicited substantially higher numbers of Rel_Mtb_-specific, IFN-γ-producing CD4+ and CD8+ T cells (P<0.0001 and P<0.0001, respectively; **Figures 2A, B**), TNF-α-producing CD4+ T cells (P=0.0076, **Figure 2C**), and IL-2-producing CD4+ and CD8+ T cells (P=0.005 and P<0.0001, respectively; **Figures 2E, F**) in the spleens of *Mtb*-infected mice, indicating that the fusion vaccine elicits more robust systemic Th1 responses compared to the comparator vaccine.

In an independent immunogenicity study using uninfected animals (**Supplementary Figure 2**), we also tested IFN-γ secretion in mouse spleens using Fluorospot (**Supplementary Figures 2B, C**). We found significantly higher secretion of Rel_Mtb_-specific IFN-γ in the spleens of mice receiving IM delivery of the IM fusion vaccine relative to those receiving IM delivery of the comparator vaccine (P=0.036). We also tested the T-cell responses in additional murine tissues, including PBMCs, across the experiment. The percentage of TNF-α-producing CD4+ T cells in the PBMC population starting at day 28 (P=0.036) and peaking at day 42 (P=0.018) after primary vaccination was significantly higher in the group receiving IM vaccination with the fusion vaccine *vs.* that receiving IM vaccination with the comparator vaccine (**Supplementary Figure 2D**).

In summary, IM delivery of the fusion vaccine demonstrated enhanced adjuctive mycobactericidal activity compared to IM delivery of the comparator vaccine and the former vaccination strategy was associated with increased systemic Th1 cytokines.

### Intranasal delivery of the *MIP-3α/rel_Mtb_* fusion vaccine showed the greatest mycobactericidal activity in combination with INH, eliciting both robust local and systemic Th1/Th17 responses in a murine model of chronic TB

**Figure 3:**
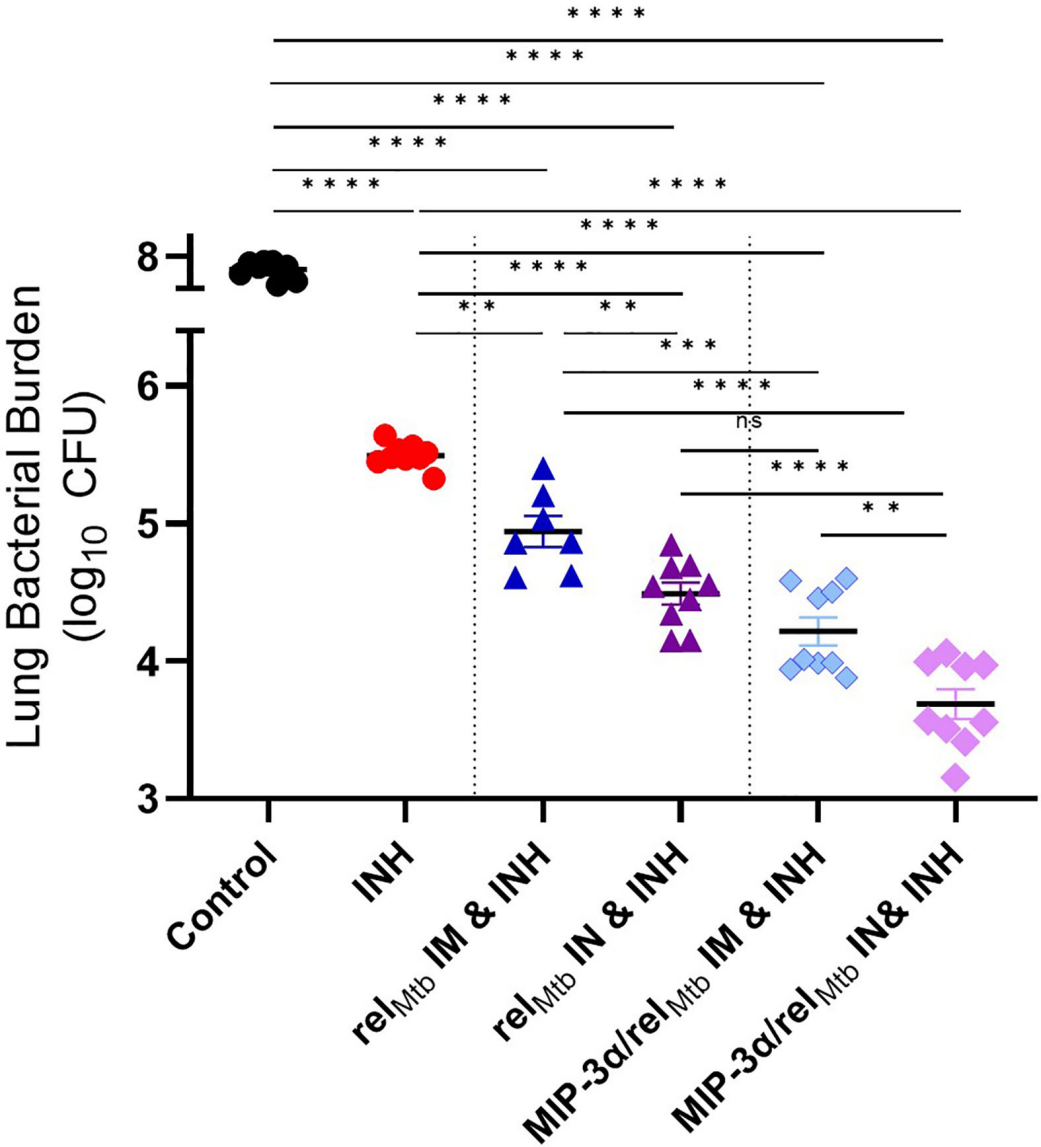
Fusion of *rel_Mtb_* with *MIP-3α* and intranasal (IN) delivery increase the mycobactericidal activity of INH in a murine model of chronic TB. The greatest therapeutic effect is demonstrated after IN delivery of the *MIP-3*α*/rel_Mtb_* fusion vaccine. Scatterplot of lung mycobacterial burden at 10 weeks after the primary vaccination per vaccination group: *Mtb*, *Mycobacterium tuberculosis*; IM, Intramuscular; IN, Intranasal; CFU, colony-forming units; INH, Isoniazid. All statistically significant P values are available in **Supplementary Table 1**. **P < 0.01, ***P < 0.001, ****P < 0.0001, ns, non-statistically significant.

Next, we investigated to what extent the IN route of vaccination could further enhance the adjuctive therapeutic activity of each vaccine compared to IM delivery. IN vaccination with the *rel_Mtb_* vaccine significantly enhanced the mycobactericidal activity of INH compared to IM vaccination with the same vaccine (“comparator vaccination strategy”) (absolute reduction of mycobacterial burden: 0.52 log_10_ CFU (P=0.0052); **Figure 3**]. IN vaccination with the *MIP-3α/rel_Mtb_* fusion vaccine (hereafter referred to as the “optimized vaccination strategy”) showed the greatest additive therapeutic effect in combination with INH relative to any other experimental group (**Figure 3**). The optimized vaccination strategy resulted in an absolute reduction in lung bacillary load of: 1.81 log_10_ relative to the INH group, 1.13 log_10_ relative to the comparator vaccination strategy (P<0.0001); 0.5 log_10_ relative to IM delivery of the *MIP-3α/rel_Mtb_* fusion vaccine (P=0.0058; **Figure 3**); and 0.61 log_10_ relative to IN delivery of the *rel_Mtb_* vaccine (P<0.0001; **Figure 3**). At 10 weeks post-primary vaccination, the optimized vaccination strategy resulted in the greatest reduction in normalized mean lung weight, which serves as a proxy for total lung inflammation, relative to the INH only group (relative reduction in normalized lung weight= 42.4%; P<0.0002) and untreated control group (relative reduction in normalized lung weight= 66.3%; P<0.0001) (**Supplementary Figure 1A**). All the individual comparisons among the different experimental groups with respect to study endpoints are listed in **Supplementary Table 1**. Mean lung mycobacterial burdens at implantation (-4 weeks), initiation of treatment (0 weeks), and at 6 weeks and 10 weeks after the initiation of treatment for each experimental group are shown in **Supplementary Figure 1B**. Gross pathology photographs of representative lungs per experimental group are available in **Supplementary Figure 1C**.

**Figure 4:**
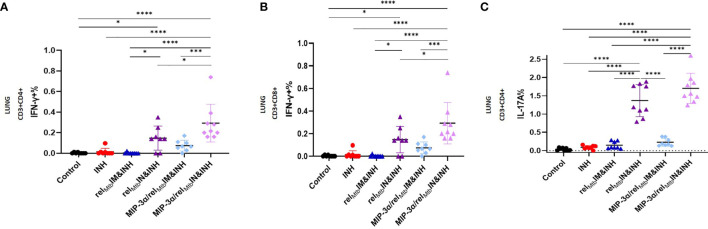
IN vaccination with the *MIP-3α/rel_Mtb_* fusion vaccine yields the most robust local Th1 and Th17 responses compared to all experimental groups. Rel*_Mtb_*-specific IFN-γ-producing CD4+ T cells **(A)** and CD8+ T cells **(B)**, Rel*_Mtb_*-specific, IL-17A-producing CD4+ T cells **(C)** as assessed by flow cytometry. IM, Intramuscular; IN, Intranasal. Y-axis scales are different among cytokines and between tissues in order to better demonstrate differences between groups where cytokine expression levels were lower. All statistically significant P values are available in **Supplementary Table 2**. *P < 0.05, ***P < 0.001, ****P < 0.0001.

Having established that IM vaccination with the *MIP-3α/rel_Mtb_* fusion vaccine induces enhanced systemic Th1 responses relative to the comparator vaccine (**Figure 2**), we next sought to investigate the effect of IN delivery of each vaccine on immune responses in the lungs, i.e., at the point of entry of *Mtb*. The optimized vaccination strategy group induced substantially greater numbers of Rel*_Mtb_*-specific, IFN-γ-producing CD4+ and CD8+ T cells (P=0.003 and P<0.0001, respectively; **Figures 4A, B**) and IL-17A-producing CD4+ T cells (P<0.0001; **Figure 4C**) in the lungs of *Mtb-*infected mice compared to IM delivery of the fusion vaccine.

**Figure 5:**
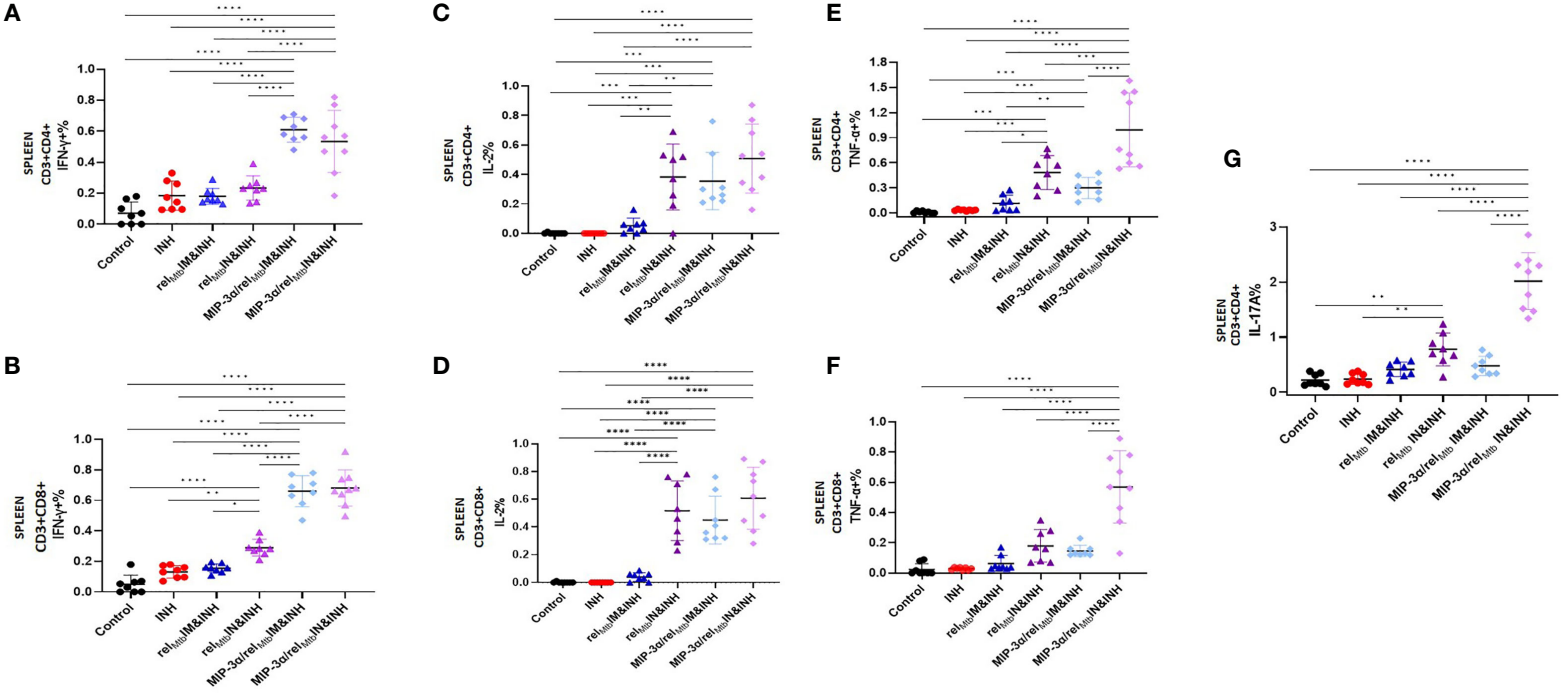
IN vaccination with the the *MIP-3α/rel_Mtb_* fusion vaccine yields robust systemic Th1 responses. Rel*_Mtb_*-specific IFN-γ-producing CD4+ T cells **(A)** and CD8+ T cells **(B)**, Rel_Mtb_-specific IL-2-producing CD4+ T cells **(C)** and CD8+ T cells **(D)**,Rel_Mtb_-specific TNF-α-producing CD4+ T cells **(E)** and CD8+ T cells **(F)**, Rel*_Mtb_*-specific, IL-17A-producing CD4+ T cells **(G)** as assessed by flow cytometry. IM, Intramuscular; IN, Intranasal. Y-axis scales are different among cytokines and between tissues in order to better demonstrate differences between groups where cytokine expression levels were lower. All statistically significant P values are available in **Supplementary Table 2**. *P < 0.05, **P < 0.01, ***P < 0.001, ****P < 0.0001.

In the context of superior local production of Th-1 and Th-17 pathway-related cytokines induced by the optimized vaccination strategy, we proceeded to compare the effect of IN delivery of the fusion vaccine on systemic *Mtb*-protective Th1 responses relative to IM administration of the same vaccine. We found that the IN delivery of the fusion vaccine (optimized vaccination strategy) elicits similarly high levels of Rel*_Mtb_*-specific, IFN-γ-producing CD4+ and CD8+ T cells (**Figures 5A, B**) and IL-2-producing CD4+ and CD8+ T cells (**Figures 5C, D**) in *Mtb*-infected spleens compared to the IM delivery of the same vaccine. Of interest, the percentage of Rel*_Mtb_*-specific, TNF-α-producing CD4+ and CD8+ T cells derived from spleens was significantly higher following IN *vs*. IM delivery of the fusion vaccine (P<0.0001 and P<0.0001, respectively; **Figures 5E, F**). Furthermore, the number of IL-17A-producing CD4+ T cells in the spleens of mice receiving IN vaccination with the fusion vaccine was also significantly higher compared to those of mice receiving the IM vaccination with the same vaccine (P<0.0001; **Figure 5G**).

In an independent immunogenicity study using uninfected animals (**Supplementary Figure 2A**), we also tested the Rel*_Mtb_*-specific, IL-17A secretion in mouse spleens using Fluorospot (**Supplementary Figures 3A, B**). We confirmed the significantly higher production of the Rel_Mtb_-specific IL-17A in the tissues of mice vaccinated with the optimized vaccination strategy compared to those receiving the fusion vaccine *via* the IM route (P=0.0079). The percentage of Rel*_Mtb_*-specific, IL-17A-producing CD4+ T cells in the PBMCs was significantly higher in the optimized vaccination strategy group *vs.* the IM fusion vaccination group (**Supplementary Figure 3C**). We also tested the T-cell responses in additional murine tissues, including draining LNs (inguinal *vs.* mediastinal). The percentage of Rel*_Mtb_*-specific, IL-17A-producing CD4+ T cells in the LNs of mice vaccinated with the optimized vaccination strategy was significantly higher compared to those vaccinated with the fusion vaccine *via* the IM route (**Supplementary Figure 3D**).

**Figure 6:**
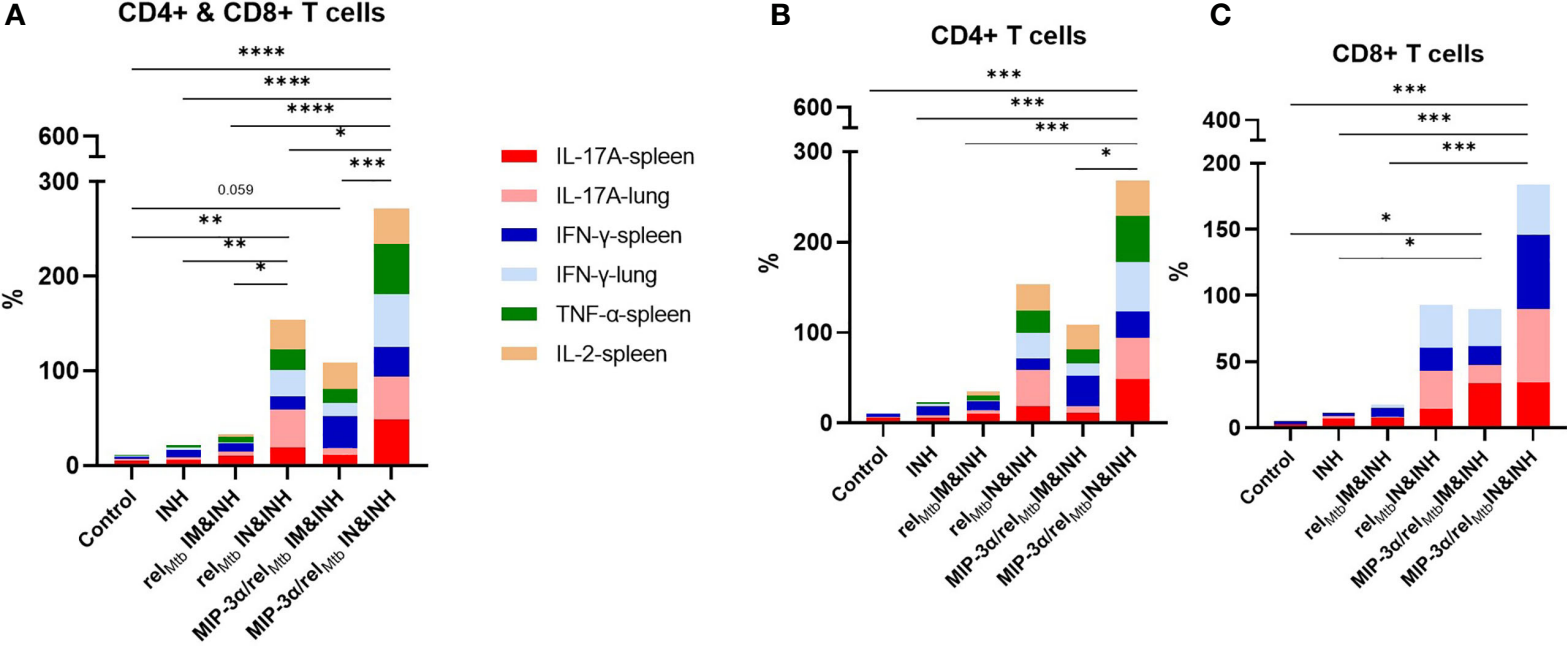
IN vaccination with the *MIP-3α/rel_Mtb_* fusion vaccine increases the simultaneous production of multiple cytokines associated with *Mtb* control, systemically and at the site of infection. Aggregate production in stack bars of IL17-A, TNF-α, IFN-γ, or IL-2-producing CD4+ and CD8+ T cells in the spleens and lungs of *Mtb*-infected animals in total CD3 population **(A)**, in CD4+ **(B)** and in CD8+ **(C)** subpopulations. *P < 0.05, **P < 0.01, ***P < 0.001, ****P < 0.0001.

Taken together, the optimized vaccination strategy, which was shown to have the most favorable microbiological outcomes relative to any other tested vaccination approach (**Figure 3**), was also found, in parallel, to be associated with significantly increased production of multiple cytokines associated with *Mtb* control, both systemically and locally in the lungs, i,e., at the site of infection (**Figures 4** and **5**). More specifically, compared to any other approach, the aggregate percentage of Rel*_Mtb_*-specific CD4+ and CD8+ T cells producing IL-17A, TNF-α, IFN-γ, or IL-2 in the spleens and lungs of *Mtb*-infected animals was significantly greater in the recipients of the intranasally administered fusion vaccine (**Figures 6A–C**, **Supplementary Table 2**).

## Discussion

The development of novel immunotherapeutic regimens that synergize with antibiotics to accelerate curative TB treatment is an attractive strategy for improving medical adherence and treatment completion rates, and for reducing costs (25). In the current study, we show that *MIP-3α* fusion and the IN route of delivery individually enhance the therapeutic adjunctive activity of a DNA vaccine targeting an *Mtb* persistence antigen in a murine model of chronic TB. Importantly, the combined, optimized approach, i.e., IN immunization with a DNA fusion vaccine expressing *MIP-3α/rel_Mtb_*, was accompanied by additive Th1/Th17 responses, both systemically and at the site of infection. This novel optimized vaccination strategy may be a promising adjunctive therapeutic approach in combination with standard anti-TB therapy.

Although a functional immunological signature predictive of adequate TB control is still lacking, it is clear that CD4+ and CD8+ T cells are critical in developing immunity against *Mtb* (26–30). T-cell immunity to TB is likely mediated by a variety of T cells, especially those mediating Th1 and Th1/Th17-like responses (31). Chronic antigenic stimulation drives antigen-specific CD4+ T-cell functional exhaustion during murine *Mtb* infection (32), with important implications for TB vaccine design. Thus, subdominant *Mtb* antigens during chronic *Mtb* infection, including Rel_Mtb,_ which is induced during antitubercular treatment (3), may represent promising targets for therapeutic vaccines in an effort to “re-educate” the immune system to tailor host anti-TB responses. Notably, our group has also studied a therapeutic vaccine targeting ESAT-6, another key *Mtb* antigen, in two chronic TB animal models. We found that the vaccination group receiving the *rel_Mtb_* DNA vaccine and INH showed a significant reduction in mycobacterial burden in the lungs of C57BL/6 mice and guinea pigs compared to the groups receiving *esat6* DNA vaccine and INH, which did not differ from the control group (3). In the current study, we chose to focus on optimizing the efficacy of the *rel_Mtb_* DNA vaccine by boosting Rel_Mtb-_specific T-cell responses through enhanced engagement with immature DCs.

Immature DCs are critical for the activation of adaptive immunity, and, eventually, mature DCs trigger antigen-specific naïve T cells (33). Of note, only a small minority of DCs are attracted to sites of immunization (33), and, in the case of HIV and TB infections, a proportion of the attracted DCs may be dysfunctional (34). Fusion of the antigen of interest to the chemokine MIP-3α (or CCL20) targets the antigen to immature DCs (35). It has been shown that following naked DNA vaccination, epidermal cells secrete the antigen of interest-MIP-3α fusion construct (20, 36). The secreted fusion construct is taken up and internalized by skin Langerhans cells *via* the receptor for this chemokine, which is termed C-C Motif Chemokine Receptor 6 (CCR6) (36). The complex is then processed and presented in draining LNs to elicit efficient cellular and humoral responses (36). Enhanced efficacy has been shown compared to antigen-only vaccines in various systems (20–22). In a mouse melanoma model, our group has demonstrated that IM immunization with a DNA vaccine containing a fusion of *MIP-3α* with the tumor antigen gene *gp100/Trp2* elicited greater numbers of tumor antigen-specific T cells and offered greater therapeutic benefit compared to the cognate vaccine lacking the *MIP-3α* fusion (20, 21). Importantly, MIP-3α has also been shown to play a key role in driving DC recruitment to the nasal mucosa (37). Indeed, we found that IM vaccination with the *MIP-3α* fusion construct corresponded to increased antigen-specific systemic Th1 responses (IFN-γ, TNF-α, Il-2 in the spleens and TNF-α in PBMCs), relative to the *rel_Mtb_* alone construct. Interestingly, although this vaccination strategy, i.e., IM vaccination with the *MIP-3α/rel_Mtb_*, fusion construct, yielded improved microbiological outcomes when combined with INH compared to the non-fused *rel_Mtb_* vaccine, no Th1 response was observed in the lungs, the primary site of the infection.

Compelling evidence suggests that protection against respiratory pathogens, such as *Mtb*, is dependent on the presence of pathogen-specific immune cells at the primary site of infection (25, 38, 39). Pre-clinical studies have shown that parenteral immunization with TB vaccines can drive robust antigen-specific T-cell responses in the periphery, but these cells are unable to rapidly enter the restricted lung mucosal compartments and largely fail to restrict *Mtb* replication (40). In contrast, respiratory mucosal immunization generates a long-lasting population of tissue-resident T cells expressing homing molecules to allow preferential migration and residence in the airway lumen and lung parenchyma (39–41). These immune cells generate an important line of defense by establishing pathogen-specific immunity at the site of entry, providing markedly enhanced control against pulmonary *Mtb* infection (39–41). Importantly, after IN vaccination, these antigen-experienced, lung-resident, T-cells have been shown to produce IL-17A in addition to IFN-γ, expanding the known signature panel that may confer enhanced TB immunity (42–45). In the current study, we have shown for the first time that IN delivery of a vaccine expressing *rel_Mtb_* enhances the bactericidal activity of an antitubercular drug (INH) relative to IM delivery of the same vaccine. Importantly, the optimized approach of *MIP-3α* fusion and the IN route of immunization yielded the greatest additive adjunctive mycobactericidal activity with INH in murine lungs, resulting in an approximate 100-fold reduction in lung bacterial burden compared to INH alone. The optimized approach was associated with more robust Th1 (IFN-γ, TNF-α, IL-2) and Th17 responses (IL-17A) systemically (spleens and PBMCs), but also in the lungs and draining LNs (IFN-γ and IL-17A), the primary site of infection.

There are limitations in our study. Although our cytokine analysis allows us to make some initial associations between each vaccination strategy and T-cell responses, future studies are needed to elucidate in detail the mechanism of the tested therapeutic vaccines, including the contribution of B cells. Also, additional studies are needed to test the therapeutic efficacy of these vaccines in other animal models which more closely represent human TB pathology, such as guinea pigs and non human primates (46).

In conclusion, we have shown that IN immunization with a DNA vaccine expressing *MIP-3α/rel_Mtb_* generates strong, additive Th1 and Th17 responses and significantly potentiates the mycobactericidal activity of the first-line drug, INH. Further studies are required to elucidate the relative importance of the different effector mechanisms elicited by this immunization strategy and to refine our understanding of the host-pathogen interactions that result in the improved therapeutic effects. Other formulations of this vaccine construct, including RNA platform, will be also the subject of future studies. Ultimately, the potential utility of this vaccination combination strategy must be evaluated as an adjunctive therapeutic intervention in shortening the duration of curative treatment for active TB in relevant preclinical models.

## Data availability statement

The original contributions presented in the study are included in the article/**Supplementary Material**. Further inquiries can be directed to the corresponding authors.

## Ethics statement

The animal study was reviewed and approved by Johns Hopkins University Institutional Animal Care and Use Committee.

## Author contributions

SKa, RBM and PCK conceived and designed the study and wrote the manuscript. JTG, AK and SKa performed the fusion vaccine construction. SKa performed transfection of 293T cells and ran western blots in lysates and supernatants. SKa and KC performed RelMtb protein expression, purification and verification. SKa performed growth, selection and extraction of vaccination plasmids. SKa and TK performed the in vivo vaccinations (immunogenicity and challenge study). SKa, PN and TK administered daily INH treatments to mice (challenge study). SKa performed survival mouse tail bleeds weekly and isolated PBMCs for further analysis (immunogenity study). SKa, PN, TK, JTG, JRC, DQ, KC, AS, YH, SKr, and CD performed animal harvesting and subsequent animal tissue processing (immunogenicity and challenge study). PN,SKr, RT and SC contributed to the in vivo challenge study design. SKa, TK and PN plated the homogenates of the infected lungs and performed colony-forming units counting and analysis. SKa and TK performed flow cytometry analysis and FluoroSpot. SKa, TK, and TW performed data and statistical analysis. SKa, JTG, PN, TK, JRC, DQ, KC, AS, AK, YH, SKr, RT, SC, CD, TW, CS, RBM and PCK interpreted the data and edited the manuscript. All authors contributed to the article and approved the submitted version.

## Funding

This work was supported by NIH grants: R21AI140860 and R01AI148710 to RM and PK, T32 AI007291 and Potts Memorial Foundation Award to SK. The content is solely the responsibility of the authors and does not necessarily represent the official views of the National Institutes of Health.

## Acknowledgements

Content of this publication has been available online in Biorxiv preprint server (47). Illustrations were created with BioRender.com.

## Conflict of interest

The authors declare that the research was conducted in the absence of any commercial or financial relationships that could be construed as a potential conflict of interest.

## Publisher’s note

All claims expressed in this article are solely those of the authors and do not necessarily represent those of their affiliated organizations, or those of the publisher, the editors and the reviewers. Any product that may be evaluated in this article, or claim that may be made by its manufacturer, is not guaranteed or endorsed by the publisher.
