# Supplementary material for: An intranasal stringent response vaccine targeting dendritic cells as a novel adjunctive therapy against tuberculosis
Source: Front Immunol. 2022 Sep 16;13:972266. doi: 10.3389/fimmu.2022.972266 (PMC9523784; doi:10.3389/fimmu.2022.972266)
Supplement: Supplementary file 1 [file DataSheet_1.docx]

**Supplementary Figure Legends**

**Supplementary Figure 1.** (A) Normalized mean mouse lung weights at 10 weeks (B) Lung mycobacterial burden at: implantation (-4 weeks); initiation of treatment (0 weeks); and at 6 weeks and 10 weeks after the initiation of treatment. (C) Gross pathology of representative lungs per experimental group; black line represents 1 cm, TB: tuberculosis, IM: Intramuscular, IN: Intranasal, CFU: colony-forming units.

**Supplementary Figure 2. Cytokine production in non-infected murine tissues 6 weeks after prime vaccination (immunogenicity study): IM vaccination with *MIP-3α/rel_Mtb_* elicited higher level of Rel_Mtb_-specific Th1 cytokines systemically compared to IM vaccination with *rel_Mtb._***(A) Timeline of the immunogenicity study. (B) Rel_Mtb_-specific IFN-γ response in spleens between IM vaccination with *MIP-3α*/*rel_Mtb_ vs rel_Mtb_* , as assessed by FluoroSpot (C) Representative pictures of FluoroSpot are shown per group. Each triplicate represents three different mice. (D) Rel_Mtb_-specific TNF-α producing CD4+ and CD8+ T cells in cells derived from PBMCs between IM *MIP-3α*/*rel_Mtb_ vs* IM *rel_Mtb_* as assessed by flow cytometry. SFU: Spot Forming Unit, IM: Intramuscular, PBMCs: Peripheral Blood Mononuclear Cells. Y-axis scales are different among cytokines and between tissues in order to better demonstrate differences between groups where cytokine expression levels were lower.

**Supplementary Figure 3: Cytokine production in non-infected murine tissues 6 weeks after prime vaccination (immunogenicity study): IN vaccination with *MIP-3α/rel_Mtb_* elicited higher levels of Rel_Mtb_-specific Th17 cytokines locally and systemically compared to IM vaccination with *MIP-3α/rel_Mtb._***(A) Rel_Mtb_-specific IL-17A response in spleens among IN *MIP-3α*/*rel_Mtb,_* IM *MIP-3α*/*rel_Mtb_* and IM *rel_Mtb_*, as assessed by FluoroSpot (B) Representative pictures of FluoroSpot are shown per group. Each triplicate represents three different mice. (C) Rel_Mtb_-specific IL-17A producing CD4+ T cells in cells derived from PBMCs as assessed by flow cytometry. (D) Rel_Mtb_-specific IL-17A producing CD4+ T cells in cells derived from draining lymph nodes as assessed by flow cytometry. SFU: Spot Forming Unit, IM: Intramuscular, IN: Intranasal, PBMCs: Peripheral Blood Mononuclear Cells, draining Lymph Nodes: mediastinal *vs.* inguinal. Y-axis scales are different among cytokines and between tissues in order to better demonstrate differences between groups where cytokine expression levels were lower.

**Supplementary Table 1.** Lung mycobacterial burden of *Mtb*-infected mice at 10 weeks post-infection. INH: Isoniazid, IM: Intramuscular, IN: Intranasal

| **LUNG MYCOBACTERIAL BURDEN** | |
| --- | --- |
| **Comparisons** | **Adjusted P Value** |
| Control vs. INH | <0.0001 |
| \| Control vs. rel*_Mtb_* IM & INH \| \| --- \| | <0.0001 |
| \| Control vs. rel_Mtb_ IN & INH \| \| --- \| | <0.0001 |
| \| Control vs. MIP-3α/rel_Mtb_ IM & ΙΝΗ \| \| --- \| | <0.0001 |
| \| Control vs. MIP-3α/rel_Mtb_ IN & ΙΝΗ \| \| --- \| | <0.0001 |
| \| INH vs. rel_Mtb_ IM & INH \| \| --- \| | \| 0.0081 \| \| --- \| |
| \| INH vs. rel_Mtb_ IN & INH \| \| --- \| | <0.0001 |
| \| INH vs. MIP-3α/rel_Mtb_ IM & ΙΝΗ \| \| --- \| | <0.0001 |
| \| INH vs. MIP-3α/rel_Mtb_ IN & ΙΝΗ \| \| --- \| | \| <0.0001 \| \| --- \| |
| \| rel_Mtb_ IM & INH vs. rel_Mtb_ IN & INH \| \| --- \| | 0.0052 |
| \| rel_Mtb_ IM & INH vs. MIP-3α/rel_Mtb_ IM & ΙΝΗ \| \| --- \| | 0.0002 |
| \| rel_Mtb_ IM & INH vs. MIP-3α/rel_Mtb_ IN & ΙΝΗ \| \| --- \| | <0.0001 |
| \| rel_Mtb_ IN & INH vs. MIP-3α/rel_Mtb_ IM & ΙΝΗ \| \| --- \| | 0.3957 |
| \| rel_Mtb_ IN & INH vs. MIP-3α/rel_Mtb_ IN & ΙΝΗ \| \| --- \| | <0.0001 |
| \| MIP-3α/rel_Mtb_ IM & ΙΝΗ vs. MIP-3α/rel_Mtb_ IN & ΙΝΗ \| \| --- \| | 0.0063 |

**Supplementary Table 2. T-cell responses in *Mtb*-infected murine tissues 6 weeks post-treatment initiation.** INH: Isoniazid, IM: Intramuscular, IN: Intranasal.

| **T-cell responses in *Mtb*-infected murine tissues** | | | | | | | | | | |
| --- | --- | --- | --- | --- | --- | --- | --- | --- | --- | --- |
|  | SPLEEN | | | | | | | LUNG | | |
|  | IFN-γ | | TNF-α | | IL-2 | | IL-17α | IFN-γ | | IL-17α |
|  | CD4+ Τ | CD8+T | CD4+ Τ | CD8+T | CD4+ Τ | CD8+T | CD4+ Τ | CD4+ Τ | CD8+T | CD4+ T |
| **Tukey’s Multiple Comparisons Test** | **Adjusted P Value** | | | | | | | | | |
| Control vs. INH | 0.3348 | 0.2939 | >0.9999 | >0.9999 | >0.9999 | >0.9999 | >0.9999 | 0.9999 | >0.9999 | 0.9990 |
| \| Control vs. rel*_Mtb_* IM & INH \| \| --- \| | 0.3741 | 0.0838 | 0.9251 | 0.9815 | 0.9871 | 0.9949 | 0.7119 | >0.9999 | >0.9999 | 0.9616 |
| \| Control vs. rel_Mtb_ IN & INH \| \| --- \| | 0.0557 | <0.0001 | 0.0010 | 0.0972 | 0.0002 | <0.0001 | 0.0025 | 0.0447 | 0.0057 | <0.0001 |
| \| Control vs.  MIP-3α/rel_Mtb_ IM & ΙΝΗ \| \| --- \| | <0.0001 | <0.0001 | \|  \| \| --- \|   0.0002 | 0.2972 | 0.0007 | <0.0001 | 0.4208 | 0.6696 | 0.4597 | 0.7090 |
| \| Control vs.  MIP-3α/rel_Mtb_ IN & ΙΝΗ \| \| --- \| | <0.0001 | <0.0001 | <0.0001 | <0.0001 | <0.0001 | <0.0001 | <0.0001 | <0.0001 | <0.0001 | <0.0001 |
| \| INH vs. rel_Mtb_ IM & INH \| \| --- \| | >0.9999 | 0.9876 | 0.9751 | 0.9894 | 0.9856 | 0.9941 | 0.7898 | 0.9998 | >0.9999 | 0.9975 |
| \| INH vs. rel_Mtb_ IN & INH \| \| --- \| | 0.9465 | 0.0020 | 0.0020 | 0.1159 | 0.0002 | <0.0001 | 0.0037 | 0.0805 | 0.0105 | <0.0001 |
| \| INH vs.  MIP-3α/rel_Mtb_ IM & ΙΝΗ \| \| --- \| | <0.0001 | <0.0001 | 0.0002 | 0.3386 | 0.0007 | <0.0001 | 0.5046 | 0.8121 | 0.5964 | 0.8957 |
| \| INH vs.  MIP-3α/rel_Mtb_ IN & ΙΝΗ \| \| --- \| | <0.0001 | <0.0001 | <0.0001 | <0.0001 | <0.0001 | <0.0001 | <0.0001 | <0.0001 | <0.0001 | <0.0001 |
| \| rel_Mtb_ IM & INH vs.  rel_Mtb_ IN & INH \| \| --- \| | 0.9268 | 0.0123 | 0.0167 | 0.3597 | 0.0018 | <0.0001 | 0.1052 | 0.0439 | 0.0063 | <0.0001 |
| \| rel_Mtb_ IM & INH vs  . MIP-3α/rel_Mtb_ IM & ΙΝΗ \| \| --- \| | <0.0001 | <0.0001 | 0.0076 | 0.7128 | 0.0050 | <0.0001 | 0.9970 | 0.6648 | 0.4816 | 0.9900 |
| \| rel_Mtb_ IM & INH vs.  MIP-3α/rel_Mtb_ IN & ΙΝΗ \| \| --- \| | <0.0001 | <0.0001 | <0.0001 | <0.0001 | <0.0001 | <0.0001 | <0.0001 | <0.0001 | <0.0001 | <0.0001 |
| \| rel_Mtb_ IN & INH vs.  MIP-3α/rel_Mtb_ IM & ΙΝΗ \| \| --- \| | <0.0001 | <0.0001 | 0.5341 | 0.9921 | 0.9993 | 0.9451 | 0.2627 | 0.6451 | 0.3761 | <0.0001 |
| \| rel_Mtb_ IN & INH vs.  MIP-3α/rel_Mtb_ IN & ΙΝΗ \| \| --- \| | <0.0001 | <0.0001 | 0.0003 | <0.0001 | 0.5818 | 0.8022 | <0.0001 | 0.0340 | 0.0064 | 0.1005 |
| \| MIP-3α/rel_Mtb_ IM & ΙΝΗ vs.  MIP-3α/rel_Mtb_ IN & ΙΝΗ \| \| --- \| | 0.717 | 0.9929 | <0.0001 | <0.0001 | 0.3635 | 0.2645 | <0.0001 | 0.0003 | <0.0001 | <0.0001 |

**Supplementary Appendix**

DNA SEQUENCE

Mouse Codon Optimized MIP-3α/Rel*_Mtb_*

Top Strand Bases

GCCGCTAGCAACTTCGACTGCTGTCTGGGATACACAGATAGAATCCTGCACCCAAAGTTCATCGTGGGCTTTACCAGACAGCTGGCCAACGAGGGATGCGACATCAACGCTATCATCTTTCACACCAAGAAGAAGCTGAGCGTGTGCGCCAACCCCAAGCAGACATGGGTGAAGTACATCGTGCGGCTGCTGAGCAAGAAGGTGAAGAACATG **(MIP-3α)**

GGACCAGGACCTGGACCAGGACCAGGACCTCAGGCGCCGAAGAGTCTCGAGgctagc **(Linker)**

ACCGCCCAGAGGTCTACCACAAACCCTGTGCTGGAGCCACTGGTGGCAGTCCACAGGGAGATCTACCCCAAGGCCGATCTGAGCATCCTGCAGAGGGCATATGAGGTGGCAGACCAGAGGCACGCCAGCCAGCTGCGCCAGTCCGGCGATCCTTACATCACACACCCACTGGCCGTGGCCAATATCCTGGCCGAGCTGGGCCTGGACACCACAACCCTGGTGGCCGCCCTGCTGCACGACACCGTGGAGGATACAGGCTATACCCTGGAGGCCCTGACAGAGGAGTTCGGAGAGGAAGTGGGACACCTGGTGGACGGAGTGACCAAGCTGGATAGGGTGGTGCTGGGCTCCGCCGCAGAGGGAGAGACAATCAGAAAGATGATCACAGCAATGGCCAGGGACCCCAGGGTGCTGGTCATCAAGGTGGCCGACCGGCTGCACAACATGAGGACCATGAGATTCCTGCCACCTGAGAAGCAGGCAAGGAAGGCCAGGGAGACACTGGAAGTGATCGCACCACTGGCCCACAGGCTGGGAATGGCCTCTGTGAAGTGGGAGCTGGAGGACCTGAGCTTTGCCATCCTGCACCCTAAGAAGTACGAGGAGATCGTGCGGCTGGTGGCAGGAAGGGCACCAAGCAGAGATACCTATCTGGCCAAGGTGCGCGCCGAGATCGTGAATACACTGACCGCCTCTAAGATCAAGGCCACAGTGGAGGGCAGGCCCAAGCACTACTGGAGCATCTATCAGAAGATGATCGTGAAGGGCAGAGACTTCGACGATATCCACGATCTGGTGGGCGTGAGAATCCTGTGCGACGAGATCCGCGATTGTTACGCAGCAGTGGGAGTGGTGCACAGCCTGTGGCAGCCAATGGCAGGCCGGTTTAAGGACTATATCGCCCAGCCCCGCTACGGCGTGTATCAGTCCCTGCACACAACCGTGGTGGGACCAGAGGGCAAGCCTCTGGAGGTGCAGATCCGGACCCGCGATATGCACAGGACAGCAGAGTACGGAATCGCAGCACACTGGAGGTATAAGGAGGCCAAGGGCAGAAACGGCGTGCTGCACCCTCACGCAGCAGCAGAGATCGACGATATGGCCTGGATGAGGCAGCTGCTGGACTGGCAGAGGGAGGCAGCCGATCCCGGAGAGTTCCTGGAGTCTCTGCGCTACGACCTGGCCGTGCAGGAGATCTTCGTGTTTACCCCTAAGGGCGACGTGATCACACTGCCCACCGGCAGCACACCTGTGGATTTTGCCTATGCAGTGCACACAGAAGTGGGACACAGGTGCATCGGAGCCCGGGTGAACGGCCGCCTGGTGGCCCTGGAGCGCAAGCTGGAGAATGGCGAGGTGGTGGAGGTGTTTACCAGCAAGGCACCAAACGCAGGACCCTCCAGAGACTGGCAGCAGTTCGTGGTGTCCCCAAGGGCCAAGACCAAGATCAGACAGTGGTTTGCCAAGGAGAGGAGAGAGGAGGCCCTGGAGACAGGCAAGGATGCCATGGCCCGGGAGGTGCGGAGGGGAGGCCTGCCCCTGCAGCGCCTGGTGAATGGAGAGTCTATGGCAGCAGTGGCCAGGGAGCTGCACTACGCAGACGTGAGCGCCCTGTATACCGCAATCGGAGAGGGACACGTGTCCGCCAAGCACGTGGTGCAGAGACTGCTGGCCGAGCTGGGAGGAATCGATCAGGCCGAGGAGGAGCTGGCCGAGAGGTCTACCCCAGCCACAATGCCCAGGAGGCCCAGATCTACCGACGATGTGGGCGTGAGCGTGCCAGGAGCACCAGGCGTGCTGACCAAGCTGGCCAAGTGCTGTACACCAGTGCCCGGCGACGTGATCATGGGATTCGTGACAAGGGGCGGAGGCGTGTCCGTGCACAGAACCGATTGTACAAACGCAGCCTCTCTGCAGCAGCAGGCAGAGAGGATCATCGAGGTGCTGTGGGCCCCTTCCCCAAGCTCCGTGTTTCTGGTGGCCATCCAGGTGGAGGCCCTGGACAGGCACAGACTGCTGTCTGATGTGACCAGAGCCCTGGCCGACGAGAAAGTGAATATCCTGTCTGCCAGCGTGACAACCTCCGGCGACAGGGTGGCCATCAGCAGGTTCACCTTCGAGATGGGCGATCCTAAGCACCTGGGCCACCTGCTGAACGCCGTGAGGAATGTGGAGGGCGTGTACGACGTGTATAGAGTGACCTCCGCCGCC **(Mouse codon optimized Rel_Mtb_)**

DNA SEQUENCE

Rel*_Mtb_* (non-codon optimized)

Top Strand Bases

GTGGCCGAGGACCAGCTCACGGCGCAAGCGGTTGCACCGCCCACGGAGGCTTCTGCGGCTCTCGAGCCCGCTCTCGAGACGCCCGAGTCGCCGGTCGAGACTCTTAAGACCAGCATCAGCGCGTCGCGTCGGGTGCGGGCCCGATTGGCCCGGCGGATGACCGCCCAGCGCAGCACCACCAATCCGGTGCTCGAGCCGTTGGTGGCGGTGCACCGGGAGATCTATCCCAAGGCCGACCTGTCGATCTTGCAGCGAGCCTACGAGGTCGCTGACCAAAGGCATGCCAGCCAGTTGCGGCAGTCCGGTGATCCCTACATCACCCACCCGTTGGCCGTTGCCAACATTCTGGCCGAGTTGGGCATGGACACCACCACTTTGGTGGCCGCGCTGCTGCACGACACCGTCGAGGACACCGGTTACACCCTGGAGGCGTTGACCGAGGAATTCGGCGAAGAGGTGGGCCATCTCGTCGACGGGGTGACCAAGCTGGATCGGGTGGTGTTGGGCAGCGCCGCCGAAGGCGAGACTATTCGCAAGATGATCACCGCGATGGCCCGCGATCCGCGGGTGCTGGTGATAAAGGTGGCTGACCGGTTACACAACATGCGCACCATGCGCTTCTTGCCGCCGGAGAAGCAGGCCCGCAAGGCCCGTGAGACGTTGGAAGTCATTGCACCCCTGGCGCATCGGCTGGGCATGGCCAGCGTCAAGTGGGAGTTGGAGGACCTGTCCTTCGCGATCCTGCATCCCAAGAAGTACGAGGAGATCGTCCGGCTGGTCGCCGGTCGCGCGCCGTCCCGGGACACCTACCTGGCCAAGGTGCGTGCCGAAATCGTCAACACGCTGACCGCGTCGAAGATCAAGGCGACGGTGGAGGGCCGCCCCAAGCACTATTGGTCGATCTACCAGAAGATGATCGTTAAGGGCCGCGACTTCGACGACATCCACGACCTGGTCGGTGTGCGCATCCTGTGCGACGAAATCCGGGACTGCTACGCGGCTGTCGGCGTAGTGCATTCGCTATGGCAGCCGATGGCGGGTCGGTTCAAGGACTACATCGCCCAGCCCAGATACGGTGTGTACCAGTCACTGCACACCACTGTGGTCGGGCCTGAGGGCAAGCCGCTGGAAGTGCAGATCCGTACCCGCGACATGCACCGCACCGCCGAATACGGCATCGCCGCGCATTGGCGCTACAAAGAAGCCAAGGGCCGCAACGGTGTTCTTCATCCGCATGCCGCCGCGGAGATCGACGACATGGCCTGGATGCGTCAGCTGCTCGACTGGCAACGTGAGGCGGCCGACCCCGGTGAGTTCTTGGAATCATTGCGCTACGACCTTGCGGTGCAAGAGATTTTCGTGTTTACCCCCAAGGGCGACGTGATCACGCTGCCAACCGGTTCGACGCCGGTGGACTTCGCTTACGCGGTGCACACAGAGGTGGGCCACCGCTGCATCGGCGCCCGAGTGAACGGCCGGTTGGTAGCGCTGGAACGCAAGCTGGAAAACGGAGAAGTTGTCGAGGTTTTCACGTCCAAGGCGCCGAACGCCGGGCCGTCGCGGGACTGGCAGCAGTTCGTGGTGTCGCCGCGCGCAAAGACGAAGATCCGCCAGTGGTTCGCCAAGGAGCGGCGTGAGGAGGCGTTGGAGACCGGTAAGGATGCGATGGCCCGCGAGGTGCGCCGCGGTGGACTTCCGTTGCAGCGCTTGGTCAATGGTGAGTCCATGGCGGCGGTGGCCCGCGAGCTGCACTACGCGGACGTGTCAGCACTCTATACCGCCATCGGTGAGGGGCACGTGTCGGCGAAACACGTCGTGCAGCGGTTGTTGGCCGAGCTCGGCGGTATCGACCAGGCGGAAGAGGAACTCGCCGAGCGGTCCACGCCGGCGACCATGCCGCGGCGCCCACGCAGCACCGACGATGTCGGGGTCTCCGTCCCCGGCGCCCCGGGCGTGCTGACCAAGCTGGCCAAGTGCTGCACGCCGGTTCCGGGCGATGTGATTATGGGGTTCGTCACCCGTGGCGGCGGGGTCAGTGTGCACCGCACCGACTGCACCAACGCCGCATCGCTGCAGCAGCAGGCCGAGCGCATCATCGAGGTGCTATGGGCGCCGTCGCCGTCGTCGGTGTTTCTGGTGGCAATCCAGGTCGAGGCACTCGACCGGCACCGGCTGCTGTCGGATGTGACGCGCGCACTGGCCGACGAGAAGGTCAATATCCTGTCCGCGTCGGTCACCACTTCGGGGGACCGGGTGGCGATCAGTCGATTCACCTTCGAGATGGGTGACCCCAAGCACCTCGGGCACCTGCTCAACGCCGTCCGCAACGTCGAAGGTGTCTACGACGTCTACCGGGTGACCTCGGCCGCG
