# Supplementary material for: An intranasal stringent response vaccine targeting dendritic cells as a novel adjunctive therapy against tuberculosis
Source: Front Immunol. 2022 Sep 16;13:972266. doi: 10.3389/fimmu.2022.972266 (PMC9523784; doi:10.3389/fimmu.2022.972266)
Supplement: Supplementary file 2 [file Presentation_1.pptx]

## Slide 1
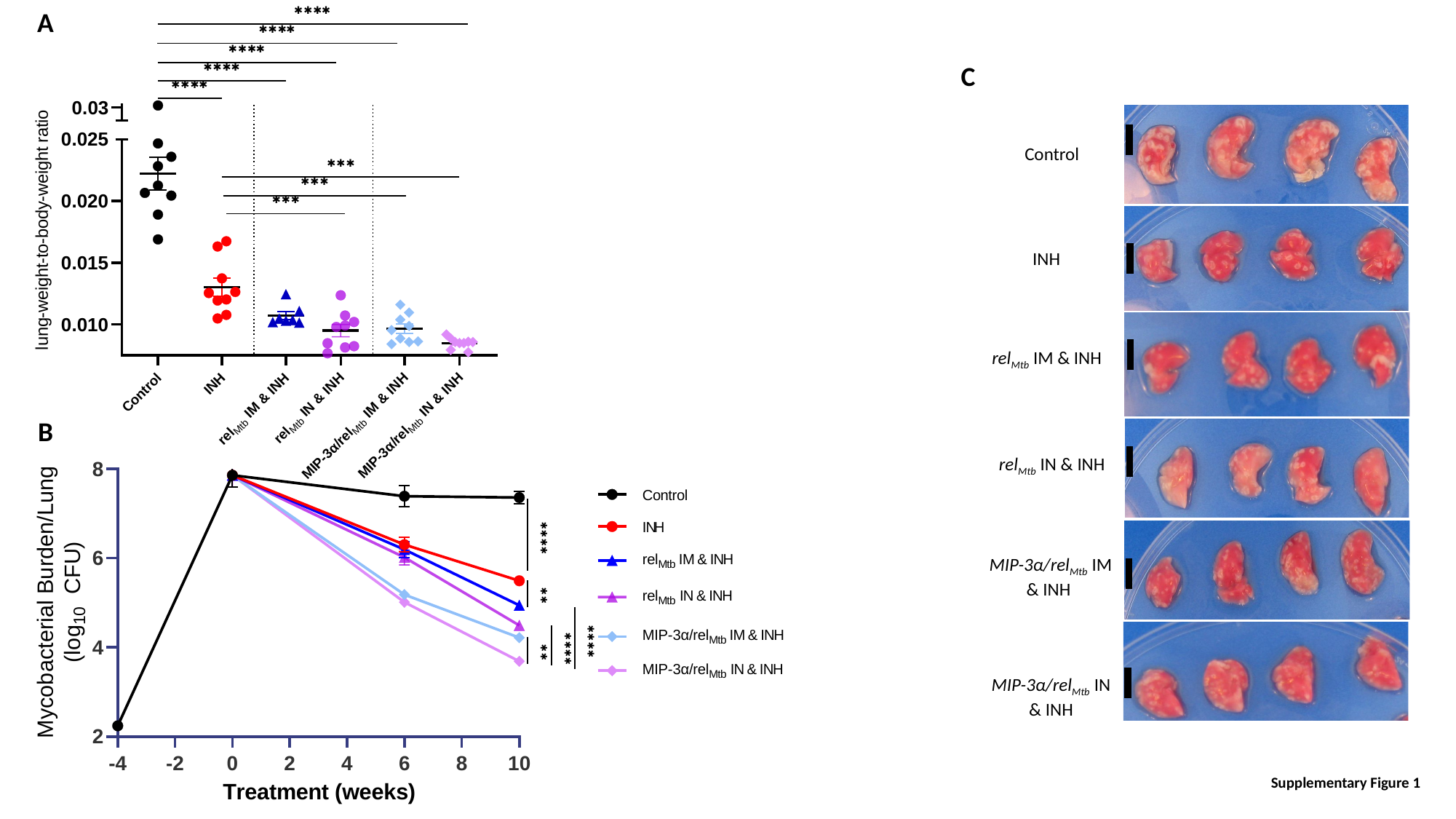

A
C
Control
 INH
relMtb IM & INH
relMtb IN & INH
MIP-3α/relMtb IM & INH
MIP-3α/relMtb IN & INH
B
Supplementary Figure 1

## Slide 2
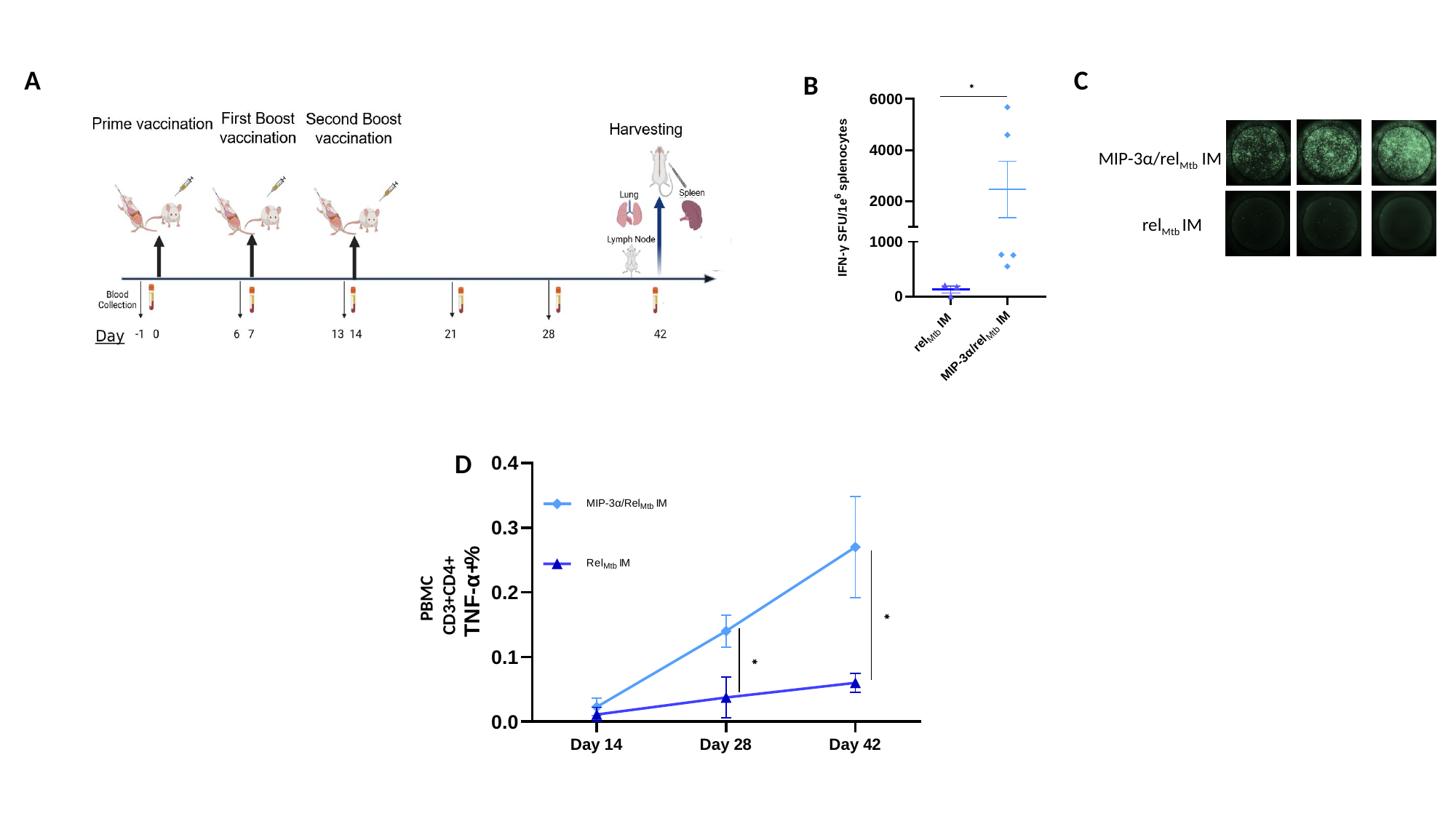

A
C
B
MIP-3α/relMtb IM
relMtb IM
D
CD3+CD4+
PBMC

## Slide 3
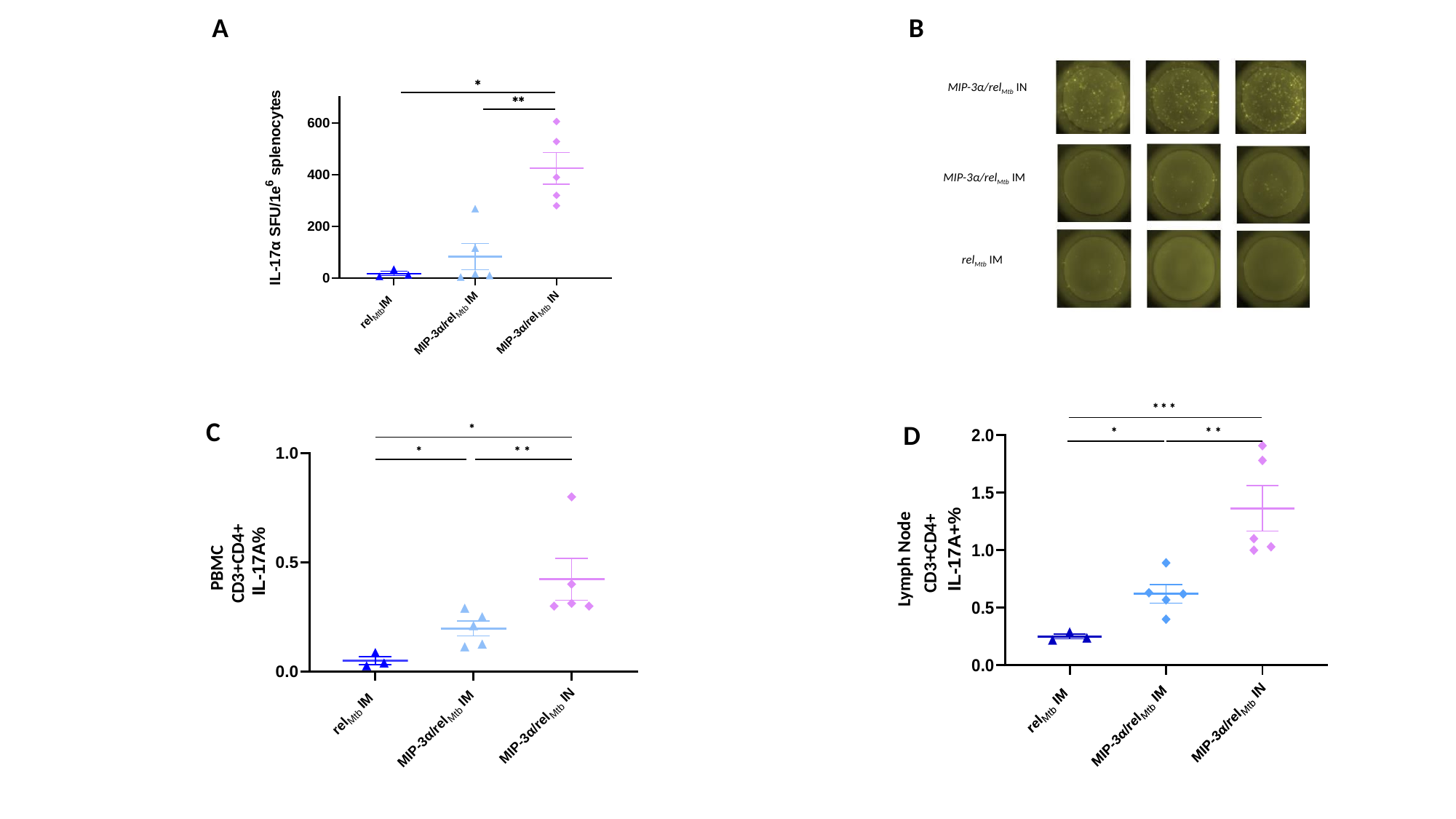

A
B
MIP-3α/relMtb IN
MIP-3α/relMtb IM
relMtb IM
CD3+CD4+
Lymph Node
D
CD3+CD4+
PBMC
C
